# Antifungal Activity of *Thapsia villosa* Essential Oil against *Candida*, *Cryptococcus*, *Malassezia*, *Aspergillus* and Dermatophyte Species

**DOI:** 10.3390/molecules22101595

**Published:** 2017-09-22

**Authors:** Eugénia Pinto, Maria-José Gonçalves, Carlos Cavaleiro, Lígia Salgueiro

**Affiliations:** 1 Laboratory of Microbiology, Biological Sciences Department, Faculty of Pharmacy of University of Porto, Rua Jorge Viterbo Ferreira n° 228, 4050-313 Porto, Portugal; 2 Interdisciplinary Centre of Marine and Environmental Research (CIIMAR/CIMAR), University of Porto, Terminal de Cruzeiros do Porto de Leixões Av. General Norton de Matos s/n, 4450-208 Matosinhos, Portugal; 3 CNC.IBILI, Faculty of Pharmacy, University of Coimbra, Azinhaga de S. Comba, 3000-354 Coimbra, Portugal; mpinho@ff.uc.pt (M.-J.G.); cavaleir@ff.uc.pt (C.C.); ligia@ff.uc.pt (L.S.)

**Keywords:** *Thapsia villosa*, essential oil, antifungal activity, inhibition yeast-mycelium transition, haemolytic activity

## Abstract

The composition of the essential oil (EO) of *Thapsia villosa* (Apiaceae), isolated by hydrodistillation from the plant’s aerial parts, was analysed by GC and GC-MS. Antifungal activity of the EO and its main components, limonene (57.5%) and methyleugenol (35.9%), were evaluated against clinically relevant yeasts (*Candida* spp., *Cryptococcus neoformans* and *Malassezia furfur*) and moulds (*Aspergillus* spp. and dermatophytes). Minimum inhibitory concentrations (MICs) were measured according to the broth macrodilution protocols by Clinical and Laboratory Standards Institute (CLSI). The EO, limonene and methyleugenol displayed low MIC and MFC (minimum fungicidal concentration) values against *Candida* spp., *Cryptococcus neoformans*, dermatophytes, and *Aspergillus* spp. Regarding *Candida* species, an inhibition of yeast–mycelium transition was demonstrated at sub-inhibitory concentrations of the EO (MIC/128; 0.01 μL/mL) and their major compounds in *Candida albicans*. Fluconazole does not show this activity, and the combination with low concentrations of EO could associate a supplementary target for the antifungal activity. The association of fluconazole with *T. villosa* oil does not show antagonism, but the combination limonene/fluconazole displays synergism. The fungistatic and fungicidal activities revealed by *T. villosa* EO and its main compounds, associated with their low haemolytic activity, confirm their potential antimicrobial interest against fungal species often associated with human mycoses.

## 1. Introduction

According to several authors, approximately 3000 plant species contain volatile oils, from which only about 100 taxa are used for essential oil production worldwide [1]. Essential oils and some of their constituents (monoterpenes, sesquiterpenes, and phenylpropanoids) are commercially relevant as perfume, cosmetic, cleaning, food, and pharmaceutical products [2]. The complexity of the composition of most essential oils, and the variety of chemical structures of their compounds, are responsible of a wide range of biological activities, with interest in the fields of human and animal health [3]. Particularly, many essential oils and their constituents have traditionally been used for their antifungal and antibacterial activities, which have long been recognized.

Fungal infections are presently considered as an important health problem around the world, and have increased intensely during the last years, due to the immunocompromised patient number. Moreover, the epidemiology has been changed with new fungal agents described as involved in systemic fungal infections [4]. Regardless, yeasts as *Cryptococcus neoformans* and *Candida* species, and filamentous fungi as *Aspergillus* species continue to be the most prevalent fungi in systemic mycosis, showing high levels of morbidity and mortality mainly in patients with compromised immunity. On the other hand, superficial cutaneous mycoses, such as mucocutaneous candidiasis, dermatophytosis, and pityriasis versicolor, remain a problem worldwide in healthy and immunocompromised patients [5].

If humans struggle to preserve their life fighting infections, microorganisms also need to keep their life by avoiding the effect of antifungal drugs used in the mycoses treatment. The emergence of drug-resistant strains to existing antifungal drugs is inevitable; we will need new antifungal drugs for treatment of infections caused by fungi with intrinsic or developed resistance to current antifungals. In addition to the resistance, other problems arise, such as a restricted number of effective antifungal compounds, and, for most of them, the fungistatic mechanism of action, high toxicity, many drug interactions, and insufficient bioavailability [6,7]. Currently, only a few antifungal agents are available for the treatment of fungal infections. Thus, effective treatments and management strategies are essential, the development of new antifungal drugs being a high priority in the healthcare field.

Apiaceae is a large plant family represented by 2500–3700 species broadly distributed worldwide. This family includes some of the most important trade essential oils, such as *Coriandrum sativum*, *Cuminum cyminum*, *Carum carvi*, *Anethum graveolens*, and *Foeniculum vulgare* [1]. Many Apiaceae species are widely used as herbal remedies, particularly as their essential oils are recognized for their antimicrobial properties [8,9,10,11,12]. *Thapsia* L. is a genus of the Apiaceae widely distributed in the Iberian Peninsula and North Africa, with some species used in folk medicine [13,14]. Also, several biological properties have been reported, such as antioxidant, antimicrobial, anti-inflammatory, and anticancer activities [14,15,16]. In the Iberian Peninsula, six *Thapsia* species have been identified: *T. garganica*, *T. transtagana*, *T. gymnesica*, *T. nitida*, *T. villosa*, and *T. minor*, with *T. villosa* and *T. minor* species being widely distributed throughout Portugal [17].

As part of our ongoing studies on the valorization of wild Apiaceae species, the aims of this study were evaluating the phytochemical constituents present in essential oil of *T. villosa* by GC and GC-MS, and determination of the antifungal activity of the essential oil and its main constituents (limonene and methyleugenol). Minimum inhibitory and fungicidal concentrations (MIC and MFC) were evaluated against fungal human pathogenic species (including clinical and ATCC isolates of *Candida* spp., *Cryptoccocus neoformans*, *Malassezia furfur*, *Aspergillus* spp., and several dermatophytes). The in vitro activity of *T. villosa*, limonene, and methyleugenol, in combination with fluconazole, was also investigated. Furthermore, the effect of the essential oil and its main constituents on the germ tube development in *C. albicans* was also studied. Evaluation of cytotoxic activity was considered using a haemolytic activity assay.

## 2. Results and Discussion

*T. villosa* collected in two regions of Portugal yielded a great amount of oils (>1.9%), thus enhancing their industrial interest. The oils were analysed by GC and GC-MS and the qualitative and quantitative compositions are presented in Table 1, where compounds are listed in order of their elution on a SPB-1 column.

High amounts of monoterpene hydrocarbons (57.4% and 58.6%) and phenylpropanoids (36.3% and 37.0%) characterised both samples. Limonene (56% and 57.5%) and methyleugenol (35% and 35.9%) were the main components identified in the two samples. Oxygen containing monoterpenes only attain 0.6% and sesquiterpenes 0.2% of the oils. Only two sesquiterpene hydrocarbons have been identified as minor components (*E*-caryophyllene and germacrene-d). These results are quite similar to those obtained previously with the oils from aerial parts of *T. villosa* from south Portugal and Spain [18], which are characterised by high amounts of methyleugenol and limonene. However, different chemical varieties have been shown in *T. villosa* oil [18,19], according to the accumulation of monoterpenes (geranyl acetate and limonene) and/or phenylpropanoids (methyleugenol and elemicin). Our samples are included in the chemotype limonene/methyleugenol. High amounts of phenylpropanoids (eugenol methyl ether) were also identified in the oil of *Thapsia transtagana* [20]. Considering the similar composition between samples A and B, sample B was used as model, and evaluated regarding biological activity.

*Thapsia villosa* essential oil (EO) (sample B) exhibited a broad-spectrum of antifungal activity (animal and human pathogenic species, causing superficial or systemic mycosis): *Candida* spp., *C. neoformans*, *M. furfur*, *Aspergillus* spp., and dermatophytes (Table 2). Minimal inhibitory concentrations of the EO were determined for the different *Candida* species and strains, and the values ranged from 0.64 to 1.25 μL/mL. No differences of behaviour were observed for fluconazole susceptible and resistant *Candida* strains. However, *C. neoformans* showed higher susceptibility to the EO, with a MIC of 0.16 μL/mL. Nevertheless, *M. furfur* showed the highest MIC value, 2.5 μL/mL. Regarding filamentous fungi, the EO showed a similar activity with a MIC of 0.64 to 1.25 μL/mL for dermatophytes and *Aspergillus* species. In addition, the EO revealed a fungicidal effect against almost all the tested fungi, with MFC values equal to, or just one log2 dilution above, the MICs. The exceptions are *A. flavus* and *A. niger*, which showed an MFC at least three times above the MIC. Concerning the major constituents of *T. villosa*, limonene and methyleugenol displayed fungicidal activity against yeasts and dermatophytes, with the MICs and MFCs ranging from 0.08 to 0.64 μL/mL. As well as for the EO, the MFCs were higher for *A. flavus* and *A. niger* for methyleugenol. Regarding methyleugenol, our results are in agreement with other previous studies that reported the fungicidal activity of this phenylpropanoid against several fungal species [21,22,23,24,25,26]. The phenylpropanoid compounds, such as eugenol and methyleugenol, are usually highly active against a broad spectrum of microorganisms, including fungi. It has been shown that they compromise the structural and functional integrity of cytoplasmic membranes, including sterol biosynthesis [21,22,26]. On the other hand, limonene shows the highest activity, being fungicidal for all the species tested, including *Aspergillus* spp. Our fungicidal activity results are in agreement with the results of fungicidal activity obtained by Chee et al. for *T. rubrum* [27]. Also, (+)-limonene showed bactericidal effect against different bacteria [28,29]. Limonene belongs to the cyclic monoterpene hydrocarbon family, being extensively used in different areas and an excellent solvent of cholesterol [30]. The hydrophobicity of limonene seems to facilitate dissolution of the lipids which are accumulated in the microbial plasma membrane, and thus, cause a loss of membrane integrity [29]. The microbial cell envelope seems to be an important target for the methyleugenol and (+)-limonene components.

Since EOs are complex natural mixtures of numerous constituents at quite different concentrations, and with possible differences in stereochemical configuration, it is difficult to attribute the antifungal activity to a specific component. Usually, the main compounds are those most responsible for the antifungal activity of the EO. However, some studies show that minor constituents may have an important role in the biological activity of the volatile oil [31]. Our results indicate that the activity of *T. villosa* EO is presumably due to the presence of methyleugenol and *(R)*-(+)-limonene (93.4% of the total composition of the EO).

**Figure 1:**
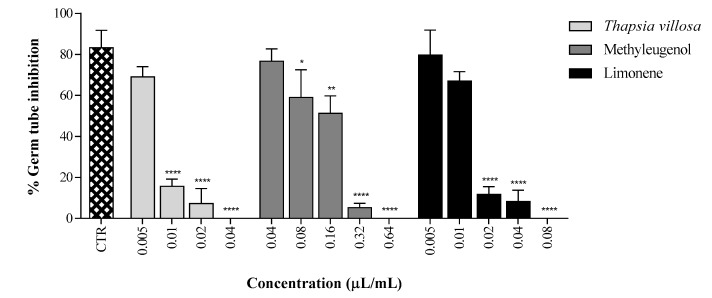
Percentage of germ tube formation by *Candida albicans* ATCC 10231 incubated with *Thapsia villosa* essential oil and its main components methyleugenol and limonene. Results are expressed as mean ± SD of three independent experiments. Statistical differences at * *p* < 0.05, ** *p* < 0.01 and **** *p* < 0.0001 (ANOVA, Tukey HSD multiple comparison test), face to the control.

Furthermore, additionally to the fungistatic or fungicidal activity, the EO of *T. villosa* showed an important inhibitory effect on germ tube development, an essential virulence factor in *C. albicans* pathogenesis [32]. The EO produced almost complete inhibition (84.3%) of filamentation in *C. albicans* ATCC 10231 at concentrations as low as MIC/128 (0.01 μL/mL), and complete inhibition (100%) at MIC/32 (0.04 μL/mL), in comparison to untreated control cells (Figure 1). The influence of the *T. villosa* EO on yeast–mycelium transition, at concentrations below the MIC (MIC/128), agreed with previous results for some EOs [33,34]. The methyleugenol and limonene were also analysed, and the activity of the EO in germ tube formation is apparently due to the involvement of the main compounds. Fluconazole did not show important inhibitory effect on germ tube formation at four times the MIC (4 μg/mL for *C. albicans* ATCC) (data not shown). The marked difference between MICs and filamentation-inhibiting concentrations is particularly relevant considering that the inhibition of the dimorphic transition in *C. albicans* has been proposed to be enough to treat candidiasis [32]. Low concentrations of *T. villosa* can affect this important mechanism of virulence for the most common agent of mucocutaneous infections. Fluconazole does not show this activity, and the combination with low concentrations of EO could associate a supplementary target for the antifungal activity. The inhibition of morphogenesis provides further validation to the potential of the EO for use alone or in combination with an antifungal compound, in therapy of candidiasis by fluconazole resistant or susceptible strains.

In synergistic assays, the EO and its major compounds (limonene and methyleugenol) were tested in combination with the antifungal, fluconazole, to assess whether there was a synergistic effect between these compounds. Fractional inhibitory concentration index (FICI) values were different according to the combination. For *T. villosa/*fluconazole and methyleugenol/fluconazole, the combination was evaluated as without interaction, showing FICI values of 3 and 0.625, respectively. However, for the combination limonene/fluconazole, the FICI value was 0.375, indicating a situation of synergism. No antagonistic effect was observed in the combination experiments with fluconazole for the tested strain. However, other reports have demonstrated a synergy or indifference between methyleugenol and fluconazole against various clinical isolates of *Candida* [22]. So, the association of fluconazole with *T. villosa* does not show antagonism, and at the same time, provides an additional target of action, by inhibition of yeast–mycelium transition. With the association, we can have (i) a fungistatic activity for fluconazole; (ii) a fungicidal activity for the essential oil at MIC concentrations; (iii) at concentrations below the MIC, a striking effect on one of the main mechanisms of pathogenicity for the most common agent of candidiasis, *C. albicans*.

On the other hand, dermatological infections have also increased in the last decades, becoming important causes of morbidity. Dermatophytoses remains a problem worldwide in healthy and immunocompromised patients. Considering the increasing impact of these infections, the emerging multidrug resistance and the toxicity of available antifungal drugs, attention has been concentrated on natural products with antifungal properties, motivating the exploration for therapeutic alternatives to conventional antifungals azoles, allylamines, and morpholinic derivatives [35]. In this study, dermatophytes showed the highest susceptibility, which can be justified by the activity observed for their two major components. Fungicidal activity is a major aspect in fungal infection treatments, once fungistatic activity can turn these infections that are refractory to therapy, resulting in important implications in health care costs, especially in poor developing countries. Also, *Malassezia furfur* is associated with several dermatological disorders, such as pityriasis versicolor, seborrheic dermatitis, and dandruff. Concerning the susceptibility of *Malassezia* species to natural antifungal agents, some studies have shown that some EOs have anti-*Malassezia* activity, like *Citrus auranifoli*, with a high content of limonene [36]. *Thapsia villosa* showed an anti-*Malassezia* activity with an MIC value of 2.5 μL/mL, higher than for dermatophytes. However, considering the fungicidal activity at the same concentration, as well the known inhibitory effect in the yeast–mycelium transition, and also a mechanism of virulence in the commensal *M. furur*, their potential in pytiriasis treatment could be considered.

Haemolytic activity was evaluated, and at 2.5 μL/mL (MICx2 at MICx4), *T. villosa*, limonene, and methyleugenol, showed 1.7%, 9%, and 1.3% of haemolysis, respectively, being indicative of a low cytotoxic activity for active antifungal concentrations. Some studies have indicated that limonene, the major component of *T. villosa* tested, does not have revealed mutagenic, carcinogenic, or nephrotoxic risk to humans, and is listed in the Code of Federal Regulations as generally recognised as safe (GRAS) for a flavouring agent [30].

Together, the fungicidal activity displayed by *T. villosa* EO, limonene, and methyleugenol, the activity against fluconazole susceptible and resistant strains, the activity against *Aspergillus* spp., and the strong ability to inhibit the germ tube formation by *C. albicans*, associated with low cytotoxic activity, confirms its potential as an antifungal agent against varied human mycoses, particularly candidiasis, cryptococcosis, and dermatophytosis. Also, the ability of EO to act on multiple cellular targets can be an advantage to avoid resistance, being active against azole resistant strains.

## 3. Material and Methods

### 3.1. Fungal Organisms

The yeasts used to evaluate the antifungal activity of the EO and its main components (limonene and methyleugenol) were *Candida* spp. clinical strains: two of *C. albicans* (M1, D5), isolated from recurrent cases of vulvovaginal and oral candidiasis; one of *C. glabrata* (D10R) isolated from recurrent case of vulvovaginal candidiasis; plus one of *C. dubliniensis* (CD1), isolated from blood; and four American Type Culture Collection (ATCC) reference strains (*C. albicans* ATCC 10231, *C. parapsilosis* ATCC 90018, *C. krusei* ATCC 6258 and *C. tropicalis* ATCC 13803). One strain of *Cryptococcus neoformans* (Colección Española de Cultivos Tipo, CECT, *C. neoformans* type strain-1078) and one clinical strain of *Malassezia furfur* (P26) were also tested. *Candida krusei* ATCC 6258 were used for quality control. Concerning the moulds, three *Aspergillus* spp. strains, *A. niger* ATCC 16404, *A. fumigatus* ATCC 46645, and an *A. flavus* strain isolated from bronchial secretions (F44), and seven dermatophyte strains, three isolated from nails and skin (*Microsporum canis* FF1, *Trichophyton mentagrophytes* FF7, and *Epidermophyton floccosum* FF9) and four from CECT collection (*M. gypseum* 2908, *T. interdigitale* 2958, *T. verrucosum* 2992, and *T. rubrum* 2794), were tested. The strains were stored in Sabouraud dextrose broth (BioMérieux, Marcy L’Etoile, France) with 20% glycerol at −70 °C. All the strains were subcultured in Sabouraud dextrose agar (SDA; BioMérieux), and SDA supplemented with olive oil for *M. furfur*, to certify optimal growth and purity, prior to use.

### 3.2. Isolation of Essential Oil from Plant Material and Reference Compounds

Aerial parts with ripe fruits of *Thapsia villosa* were collected in Central Portugal: Penacova (sample A) and Serra da Boa Viagem (sample B). Plant taxonomy was confirmed, and voucher specimens were deposited in the Herbarium of the Department of Life Sciences of the University of Coimbra (COI) and in the Herbarium of the Faculty of Pharmacy of the University of Coimbra. The essential oil was isolated by hydrodistillation for 3 h using a Clevenger-type apparatus, according to the procedure described in the European Pharmacopoeia [37]. Analytical standard of *(R)*-(+)-limonene and methyleugenol, ≥99.0% and >98% of purity respectively, were purchased from Fluka-Sigma-Aldrich (St. Louis, MO, USA).

### 3.3. Chemical Composition

#### 3.3.1. Gas Chromatography (GC)

Analytical GC was carried out in a Hewlett-Packard 6890 chromatograph (Agilent Technologies, Santa Clara, CA, USA) with a HP GC ChemStation Rev. A.05.04 data handling system, equipped with a single injector and two flame ionization detectors (FID). A graphpak divider was used for simultaneous sampling to two Supelco fused silica capillary columns: SPB-1 (polydimethylsiloxane 30 m × 0.20 mm, film thickness 0.20 μm), and SupelcoWax-10 (SW10) (polyethylene glycol 30 m × 0.20 mm, film thickness 0.20 μm). The oven temperature was programmed at 70–220 °C (3 °C/min), 220 °C (15 min); injector temperature: 250 °C; carrier gas: helium, adjusted to a linear velocity of 30 m/s; splitting ratio 1:40; detectors temperature: 250 °C.

#### 3.3.2. Gas Chromatography-Mass Spectrometry (GC-MS)

GC-MS was carried out in a Hewlett-Packard 6890 gas chromatograph fitted with a HP1 fused silica column (polydimethylsiloxane 30 m × 0.25 mm, film thickness 0.25 μm), interfaced with an Hewlett-Packard mass selective detector 5973. GC parameters as described above; interface temperature: 250 °C; MS source temperature: 230 °C; MS quadrupole temperature: 150 °C; ionization energy: 70 eV; ionization current: 60 μA; scan range: 35–350 units; scans per second: 4.51.

#### 3.3.3. Identification of Individual Components

The volatile compounds were identified based on their retention indices on both columns and through their mass spectra. Retention indices, calculated by linear interpolation relative to retention times of C_8_–C_24_ of *n*-alkanes, were compared with those of reference compounds included in FFUC laboratory database or literature data [38,39]. Acquired mass spectra were compared with reference spectra from the laboratory and literature data [38,39,40]. Relative amounts of individual components were calculated as mean values of two injections from each oil sample without using response factors.

### 3.4. Antifungal Activity

Minimum inhibitory concentrations (MICs) of the EO and its main components were determined by broth macrodilution methods based on the Clinical and Laboratory Standards Institute (CLSI) reference documents M27-A3, S3 [41], and M38-A2 [42], for yeasts and filamentous fungi, respectively, with some adaptations. Concisely, fungal organism cell suspensions from SDA cultures were prepared in RPMI-1640 medium. Considering *M. furfur*, olive oil was added to the RPMI medium. Two-fold serial dilutions of the EO and its main pure constituents (limonene and methyleugenol), ranging from 0.02 to 20 μL/mL, were prepared in DMSO (Sigma-Aldrich, St. Louis, MO, USA) with a maximum concentration of 1% (*v*/*v*). Mixed systems were incubated in humid atmosphere, without agitation, at 35 °C for 48 h for *Candida* and *Aspergillus* spp., 72 h for *C. neoformans* and *M. furfur*, and at 25 °C for seven days for dermatophytes. MIC was considered the lowest concentration resulting in 100% growth inhibition. Moreover, fluconazole (Sigma-Aldrich; 0.25 to 128 mg/L), for yeasts and dermatophytes, or amphotericin B (Sigma-Aldrich; 0.016 to 16 mg/L), for *Aspergillus*, were used as standard agents. MICs of fluconazole and amphotericin B against *C. krusei* ATCC 6258, quality control strain, were also performed and the results were within the CLSI proposed limits. Growth and sterility controls were performed in RPMI 1640 medium with or without 1% DMSO. The minimum fungicidal concentrations (MFCs) were determined after incubation time (48 h for yeasts and *Aspergillus* spp. and seven days for dermatophytes), by removing 20 μL from all tubes deprived of visible growth to SDA plates and SDA with olive oil for *M. furfur*. The plates were incubated at 25 °C for dermatophytes and at 35 °C for the other fungi. The MFC was defined as the lowest concentration showing a total absence of growth on SDA plates, resulting from the subculture of MIC plates. All the experiments were repeated at least three times, on independent assays. MIC was considered the concentration value that has been repeated at least three times and results ranging from two concentrations were shown as an interval of values.

### 3.5. Interaction Test by Checkboard Microdilution Assay

Despite its limitations, the checkerboard method remains the most popular approach for evaluating drug interactions between antifungals [43]. The MICs for different drugs, alone and in combination, were determined using the checkerboard micromethod in a 96 well plate. This assay was performed with *C. parapsilosis* ATCC 90018, by testing the synergism of the combination of *T. villosa*, limonene and methyleugenol with the antifungal fluconazole. The plates were incubated at 35 °C and the results of the MIC were read after 24 h. The results were obtained from tests repeated at least 3 times on different days. The substance combinations were analysed by calculating the FICI. It was proposed that a FICI <0.5 should be considered “synergism”, a FICI > 4 should be considered “antagonism” and a FICI between 0.5 and 4 should be considered “without interaction” [43].

### 3.6. Germ Tube Inhibition Assay

The effect of the *T. villosa* EO, limonene, and methyleugenol on the yeast–mycelium transition, was evaluated in a suspension of *C. albicans* strain ATCC 10231, prepared in NYP medium (*N*-acetylglucosamine (Sigma-Aldrich; 10^−3^ mol/L), yeast nitrogen base (Difco-Becton Dickinson, Le Pont de Claix, France; 3.35 g/L), proline (Fluka-Sigma-Aldrich; 10^−3^ mol/L), and NaCl (4.5 g/L), at pH 6.7 ± 0.1 [44]. The cell suspension, prepared from overnight cultures in SDA, was adjusted to a yeast density of 1.0 ± 0.2 × 10^6^ CFU/mL, and 990 μL volumes were distributed into glass test tubes. Dilutions of the EO, limonene, and methyleugenol, were added into the cell suspension tubes, in 10 μL volumes, to achieve appropriate MIC and sub-inhibitory concentrations. After incubation, without agitation at 37 °C for 3 h, the treated and untreated (positive control) yeast cells were observed under light microscope, and the percentage of germinating cells was determined. The germ tube formation was considered positive when the germinating tube had higher, or the same, blastospore diameter. The results show the average ± standard deviation (SD) of a minimum of three independent experiments.

### 3.7. Haemolytic Activity

Haemolytic activity was evaluated for *T. villosa* EO, limonene, methyleugenol, and amphotericin B, as a commercial antifungal drug, using human red blood cells from healthy individuals, according to the protocol described by Ahmad et al. [21].

### 3.8. Statistical Analysis

Data were analysed using GraphPad Prism 7 Software, Inc. (San Diego, CA, USA) for Windows. One-way ANOVA with Tukey’s HSD (honest significant difference), as a post hoc test, was employed to compare the percentage of germ tube formation by *C. albicans* ATCC 10231 incubated with *Thapsia villosa* essential oil and its main components, considering the respective control. Values were expressed as mean ± SD of three independent experiments.

## 4. Conclusions

These results revealed that *Thapsia villosa* yielded a great amount of oil that is a possible source of bioactive compounds with valuable antifungal activity. The wide-spectrum antifungal activity displayed by the *T. villosa* EO, and two of its main components, limonene and methyleugenol, with their associated fungicidal mechanism and low cytotoxic activity, confirms its potential as an antifungal agent against fungal species implicated in human mycoses, particularly candidiasis, cryptococcosis, and dermatophytosis. Their association with commercial antifungal compounds, such as fluconazole used in candidiasis treatment, although not showing a synergistic effect, could bring benefits based on the striking effect on germ tube formation. Further investigation will be necessary for the development of clinically useful therapeutics for clinical use in the treatment of superficial and mucosal mycoses, namely, dermatophytosis and mucocutaneous candidiasis.

## Figures and Tables

**Table 1:** Constituents of the essential oil of *Thapsia villosa* from Portugal.

Compound *	RI	RI	%	%
	SPB-1 ^a^	SW 10 ^b^	A	B
α-Thujene	922	1029	0.1	t
α-Pinene	930	1030	0.1	t
Sabinene	964	1128	0.1	t
β-Pinene	970	1118	0.1	t
Myrcene	980	1161	0.2	0.3
*p*-Cymene	1012	1272	0.3	0.3
Limonene	1020	1206	56.0	57.5
γ-Terpinene	1046	1249	0.5	0.3
Nonanal	1084	1393	0.1	t
Linalool	1084	1541	0.1	0.1
Terpinen-4-ol	1158	1597	t	t
α-Terpineol	1169	1692	t	t
*cis*-Carveol	1206	1860	0.1	0.1
Carvone	1212	1728	0.1	0.1
*E*-*p*-Menth-1(7),8-dien-2-ol	1167	1790	t	t
*Z*-*p*-Menth-1(7),8-dien-2-ol	1240	1555	t	0.1
Methyleugenol	1368	2012	35.0	35.9
*E*-Caryophyllene	1408	1590	0.1	t
Germacrene-d	1466	1699	0.1	0.1
Myristicine	1485	2253	0.1	t
Elemicine	1516	2253	1.2	1.0
Monoterpene hydrocarbons			57.4	58.6
Oxygen containing monoterpenes			0.5	0.6
Sesquiterpene hydrocarbons			0.2	0.1
Phenylpropanoids			36.3	37.0
Other compounds			0.1	t
Total identified			94.5	96.3

* Compounds listed in order of elution in the SPB-1 column. t = traces ≤ 0.05%. ^a^ RI SPB 1: GC retention indices relative to C_9_-C_23_ *n*-alkanes on the SPB-1 column. ^b^ RI SW 10: GC retention indices relative to C_9_-C_23_ *n*-alkanes on the Supelcowax-10 column.

**Table 2:** Antifungal activity (MIC and MFC) of the *Thapsia villosa* (sample B) for yeast, dermatophyte, and *Aspergillus* strains.

			Essential Oil		Methyleugenol		*(R)*-(+)-Limonene		Fluconazole		Amphotericin B	
			MIC ^a^	MFC ^a^	MIC ^a^	MFC ^a^	MIC ^a^	MFC ^a^	MIC ^b^	MFC ^b^	MIC ^b^	MFC ^b^
**Yeasts**	**ATCC**	*Candida albicans* ATCC 10231	1.25	1.25–2.5	0.64	0.64	0.32	0.32	1	>128	-	-
		*C. krusei* ATCC 6258	0.64	1.25	0.64	0.64	0.16	0.16	64	64–128	-	-
		*C. tropicalis* ATCC 13803	0.64–1.25	1.25	0.64	0.64	0.64	0.64	4	>128	-	-
		*C. parapsilosis* ATCC 90018	1.25	1.25–2.5	0.32–0.64	0.64	0.64	0.64	1	1–2	-	-
		*Cryptococcus neoformans* CECT 1078	0.16	0.16	0.32	0.32–0.64	0.08	0.08	16	128	-	-
	**Clinical isolates**	*C. albicans* D5	1.25	1.25	0.64	0.64	0.16	0.16	64	>128	-	-
		*C. albicans* M1	1.25	2.5	0.64	0.64	0.64	0.64	2	128	-	-
		*C. dubliniensis* CD1	1.25	1.25	0.64	0.64	0.16	0.16	1	>128	-	-
		*C. glabrata* D10R	1.25	2.5	0.64	0.64	0.32	0.32–0.64	32	32	-	-
		*Malassezia furfur* P26	2.5	2.5	-	-	-	-	-	-	-	-
**Filamentous fungi**	**Dermatophytes**	*Epidermophyton floccosum* FF9	0.64	0.64	0.32	0.32	0.08	0.08	16	16	-	-
		*Trichophyton rubrum* CECT 2794	0.64	0.64	0.32	0.64	0.08	0.08	16	64	-	-
		*T. mentagrophytes* FF7	0.64	1.25	0.32	0.32–0.64	0.16	0.16	16–32	32–64	-	-
		*T. mentagrophytes* var. *interdigitale* CECT 2958	1.25	1.25	0.32	0.64	0.16	0.16	128	≥128	-	-
		*T. verrucosum* CECT 2992	1.25	1.25	0.32	0.32	0.16	0.16	>128	>128	-	-
		*Microsporum canis* FF1	0.64	0.64	0.32	0.32	0.08	0.16	128	128	-	-
		*M. gypseum* CECT 2908	1.25	1.25	0.32	0.32–0.64	0.08–0.16	0.08–0.16	128	>128	-	-
	***Aspergillus*** **species**	*Aspergillus flavus* F44	1.25	>5	0.64	>2.5	0.32–0.64	0.64	-	-	2	8
		*A. fumigatus* ATCC 46645	1.25	1.25–2.5	0.32	1.25	0.32	0.32	-	-	2	4
		*A. niger* ATCC 16404	0.64	≥5	0.64	>2.5	0.32	0.64	-	-	1–2	4

**^a^** Minimum inhibitory concentration (MIC) and minimum fungicidal concentration (MFC) were determined by a macrodilution method and expressed in μL/mL (*v*/*v*). **^b^** MIC and MFC were determined by a macrodilution method and expressed in μg/mL (*w*/*v*). - Not tested. MIC was considered the concentration value that has been repeated at least three times with the same result and an interval of values were shown when the results ranging from two concentrations.
